# Chlamydiae as pathogens: new species and new issues.

**DOI:** 10.3201/eid0204.960406

**Published:** 1996

**Authors:** R W Peeling, R C Brunham

**Affiliations:** Laboratory Centre for Disease Control Health, Winnipeg, Manitoba, Canada. rosanna_peeling@isdtcp3.hwc.ca

## Abstract

The recognition of genital chlamydial infection as an important public health problem was made first by the recognition of its role in acute clinical syndromes, as well as in serious reproductive and ocular complications, and secondly by our awareness of its prevalence when diagnostic tests became widely accessible. The recent availability of effective single dose oral antimicrobial therapy and sensitive molecular amplification tests that allow the use of noninvasive specimens for diagnosis and screening is expected to have a major impact in reducing the prevalence of disease in the next decade. Clinical manifestations associated with Chlamydia pneumoniae infection continue to emerge beyond respiratory illness. In particular, its association with atherosclerosis deserves further investigation. Chlamydia pecorum, a pathogen of ruminants, was recently recognized as a new species. The continued application of molecular techniques will likely elucidate an expanding role for chlamydiae in human and animal diseases, delineate the phylogenetic relationships among chlamydial species and within the eubacteria domain, and provide tools for detection and control of chlamydial infections.


### Chlamydiae as Pathogens: New Species and New Issues

**Rosanna W. Peeling* and Robert C. Brunham†**
*Laboratory Centre for Disease Control Health, Winnipeg, Manitoba, Canada
†Department of Medical Microbiology, University of Manitoba, Winnipeg, Canada

The recognition of genital chlamydial infection as an important public health problem was made first by the recognition of its role in acute clinical syndromes, as well as in serious reproductive and ocular complications, and secondly by our awareness of its prevalence when diagnostic tests became widely accessible. The recent availability of effective single dose oral antimicrobial therapy and sensitive molecular amplification tests that allow the use of noninvasive specimens for diagnosis and screening is expected to have a major impact in reducing the prevalence of disease in the next decade. Clinical manifestations associated with *Chlamydia pneumoniae* infection continue to emerge beyond respiratory illness. In particular, its association with atherosclerosis deserves further investigation. *Chlamydia pecorum*, a pathogen of ruminants, was recently recognized as a new species. The continued application of molecular techniques will likely elucidate an expanding role for chlamydiae in human and animal diseases, delineate the phylogenetic relationships among chlamydial species and within the eubacteria domain, and provide tools for detection and control of chlamydial infections.

Chlamydiae are obligate intracellular bacteria that grow in eukaryotic cells and cause a wide spectrum of human disease (Table). Species were grouped according to their biologic and biochemical properties and a greater than 95% homology in their 16s ribosomal RNA sequences (1). Molecular analyses led to the reclassification of some *Chlamydia psittaci* strains as *Chlamydia pneumoniae*, a human pathogen, and *Chlamydia pecorum*, a pathogen of ruminants. Given the diverse host range of *C. psittaci* strains, more reclassification within this species may be likely.

The oldest reported disease associated with *C. trachomatis* infection is trachoma, a sequela of ocular infection. This disease was described in China and in the Ebers papyrus in Egypt thousands of years ago and continues to be a major cause of preventable blindness, with an estimated 500 million cases of active trachoma worldwide (seven million include blindness from conjunctival scarring and eyelid deformities [2]). In the last two decades, genital chlamydial infection has been identified as a major public health problem because of the recognition that chlamydial infection is associated with disease syndromes such as nongonococcal urethritis, mucopurulent cervicitis, pelvic inflammatory disease (PID), ectopic pregnancy, and tubal infertility. The World Health Organization estimated 89 million new cases of genital chlamydial infections worldwide in 1995 (3). In the United States, each year an estimated four million new cases occur and 50,000 women become infertile as a result of infection (4).

*C. psittaci* infection, acquired through respiratory droplet transmission of chlamydiae from infected birds, has been considered for many years an occupational hazard for employees of pet shops and poultry processing plants (5). Sources of human *C. psittaci* infection other than infected birds have been identified and may

Address for correspondence: Rosanna W. Peeling, LCDC Chlamydia Laboratory, Health Sciences Centre MS 673C, 820 Sherbrook St., Winnipeg, Manitoba, Canada R3A 1R9; fax: 204-787-4699; e-mail: rosanna_peeling@isdtcp3.hwc.ca.

Table. Spectrum of human diseases caused by Chlamydiae

| Species | Acute Diseases | Sequelae/Chronic Diseases |
|---|---|---|
| *C. trachomatis* | | |
| Serovars A-C | conjunctivitis | trachoma |
| Serovars D-K | urethritis | proctitis, epididymo-orchitis, Reiter's Syndrome |
| | cervicitis | pelvic inflammatory disease, ectopic pregnancy, tubal infertility, Fitz-Hugh Curtis Syndrome |
| | ophthalmia neonatorum | |
| | neonatal pneumonia | |
| LGV serovars | lymphogranuloma venereum | |
| *C. pneumoniae* | pharyngitis | ?cardiovascular disease |
| | sinusitis | ?asthma |
| | bronchitis | |
| | community-acquired pneumonia | |
| *C. psittaci* | | |
| parrot | atypical pneumonia | |
| canaries | hepatic and renal | |
| pigeons | dysfunction | |
| turkeys | endocarditis | |
| ducks | | |
| chickens | | |
| cats | conjunctivitis | |
| ewes | abortion | |

be more common than currently recognized. Detection of *C. psittaci* in household cats and breeding catteries illustrates the expanding number of chlamydial diseases in animals that are transmissible to humans (6,7).

*C. pneumoniae* is a human pathogen recognized as an important cause of respiratory illness (8). Approximately 40% to 60% of adult populations around the world have antibodies to *C. pneumoniae*, which suggests that the infection is extraordinarily prevalent, and reinfection is common. Current interest centers on the emerging role of *C. pneumoniae* infection in the pathogenesis of atherosclerosis and asthma.

### Biology of Chlamydiae: An Update

Chlamydiae have a unique biphasic life cycle with dimorphic forms that are functionally and morphologically distinct. An extracellular form, the elementary body (EB), is infectious but metabolically inactive. Once endocytosed, the EB differentiates into a larger pleomorphic form called the reticulate body (RB), which replicates by binary fission. The precise mechanism by which EBs attach and gain entry into the host cell is unknown. Recent work suggests that chlamydiae employ a molecular mimic of heparan sulfate to attach to glycosaminoglycan (GAG) receptors on eukaryotic cell surfaces (9). GAG appears to form a trimolecular complex with the host cell since (EB) infectivity is inhibited by the addition of heparan or heparan sulfate to culture, and pretreatment of EBs with heparan sulfate lyase abolishes EB infectivity. The mechanism of endocytic uptake remains unclear. Once inside the host cell, chlamydiae reside in a membrane-bound vacuole that can evade phagolysosomal fusion. The endosome is transported to the distal region of the Golgi apparatus and incorporates host-derived sphingolipids into the inclusion membrane (10,11). Thus it appears that chlamydiae are able to intercept host vesicular traffic bound for the plasma membrane to sequester lipids and possibly other host substances synthesized in the Golgi. Subversion of host vesicular traffic may represent a dual advantage for chlamydiae in obtaining materials from the host for its metabolism as well as in modifying the inclusion membrane to evade lysosomal fusion and immune detection.

Chlamydiae are considered energy parasites because they lack the enzymes of the electron transport chain and thus require adenosine triphosphate (ATP) and nutrient resources from the host to fuel their metabolism and replication. Chlamydiae are incapable of de novo nucleotide biosynthesis and are dependent on host nucleotide pools (12). In spite of the successful selection of various metabolic mutants of *C. trachomatis*, progress in elucidating the host-parasite metabolic relationship has been hampered by multiple salvage metabolic pathways in the host and the lack of a genetic shuttle system for chlamydiae.

### *C. trachomatis*

### Epidemiology

Genital infections due to *C. trachomatis* are the most common sexually transmitted diseases in many industrialized countries (3). Each year, an estimated four million new cases occur in the United States and three million in Europe. These infections present unique problems for public health control programs because 50 % to 70% of infections in women (and perhaps men) are clinically silent. Unrecognized and untreated, the bacteria may remain infectious in the host for months and be readily transmitted to sex partners. Furthermore, most reported infections occur in the 15- to 24-year-old age group. Young women with cervical chlamydial infections are at risk for pelvic inflammatory disease, which can lead to long-term reproductive sequelae such as chronic pelvic pain, ectopic pregnancy, and tubal infertility. Babies born to infected mothers are also at risk for conjunctivitis and pneumonia. The annual direct and indirect costs of genital chlamydial infections in the United States are estimated at $2.4 billion (4).

Control programs emphasizing early diagnosis, targeted screening, partner notification, and effective treatment have led to a slow decline in the incidence of genital chlamydial infection in countries where these programs have been implemented (13). The true rate of decline may be higher than the reported rate because of increased sensitivity of laboratory testing and more widespread screening. In women, screening of chlamydial infection at the time of Papanicolaou tests, prenatal visits, or attendance at family planning or pregnancy counseling clinics have been effective. In asymptomatic men, who are less likely to access care, asymptomatic infection is not adequately addressed by current public health programs.

In contrast to genital chlamydial infection, trachoma is a household disease that has disappeared in many parts of the world because of improved living conditions and hygiene. In trachoma-endemic areas, severe disease leading to scarring and blindness may be the result of frequent reinfection or persistent infection in those whose immune system does not mount an adequate response to clear the infection. For both ocular and genital chlamydial infections, recent advances in diagnostic and screening technology and single dose antimicrobial therapy will likely have a significant impact on the efficacy of disease control programs and the opportunity for eventual disease eradication.

### Laboratory Diagnosis

Since curative antibiotic therapy for chlamydial infections is readily available and inexpensive, early diagnosis is an essential component of public health programs to control these infections. The goals of early identification are to interrupt the chain of transmission in the community and to prevent long-term sequelae. Isolation of the organism in cell culture had been the traditional method for laboratory diagnosis and has remained the method of choice for medicolegal specimens because of its specificity. However, culture requires expensive equipment, technical expertise, and stringent transport conditions to preserve specimen viability; it also has a turnaround time of 2 to 3 days. Hence, in many settings, culture has been replaced by antigen-detection methods, such as enzyme immunoassays (EIA) and direct fluorescence assays (DFA), which have less demanding transport requirements and can provide results on the same day. EIAs are suitable for public health laboratories serving large geographic areas because specimens are stable in transport under ambient conditions and are inexpensive because they allow specimens to be processed in batches by automated equipment. Assays are typically based on the capture of the chlamydial lipopolysaccharide (LPS) using monoclonal or polyclonal antibodies linked to a solid-phase support. Early problems with low specificity because of cross-reactivity between the chlamydial LPS and that of other gram-negative bacteria have been largely overcome by confirmation with DFA or a blocking antibody assay. With a lower detection limit of 10,000 elementary bodies, EIA lacks sensitivity as a screening assay, especially for asymptomatic men (14,15). Nucleic acid-based hybridization probe tests offer higher specificity but no substantial improvement on sensitivity (15). Nucleic acid amplification tests based on polymerase chain reaction (PCR), ligase chain reaction (LCR), and transcription-mediated amplification technology are now commercially available. The precision of nucleic acid hybridization and the rapid amplification of a single gene target facilitated the design of diagnostic tests with specificities in excess of 99% and lower detection limits of 1-10 EBs. In addition, these tests offer all the advantages of nonculture tests in terms of ambient specimen transport, batching, automation, and rapid processing time of 4 hours. Duplex testing for the simultaneous detection of chlamydial and gonococcal DNA from a single specimen is also commercially available in some countries.

A major advantage of the increased sensitivity of these molecular amplification tests is that noninvasive specimens, such as urine, can be used for testing. The ease of collection and the lack of sampling bias of urine specimens make screening feasible in settings outside physicians' offices. PCR assays on urethral or cervical swabs for the laboratory diagnosis of genital chlamydial infection in symptomatic men and women show sensitivities of 89% to 100% and specificities of 99% to 100% compared with the traditional culture or PCR test, confirmed by a second PCR reaction targeting a different gene (16-18). For urine specimens, PCR assays show sensitivities of 87% to 100% for men and 92% for women and specificities of 96% to 100% for men and 95% for women (18-20). In a study of 447 women with a prevalence of infection of 6%, the sensitivity of urine LCR was 96% compared with 56% for cervical swab culture, 78% for cervical swab EIA, and 37% for urine EIA (21). For men in the same study, the sensitivity of urine LCR was 96% compared with 68% for urine EIA, and 38% for urethral swab culture. In a multicenter study of 2,132 women, cervical swab LCR showed a sensitivity of 87% to 98% compared with a sensitivity of 52% to 92% for culture (22). In LCR studies, a true positive was defined as culture positive or LCR positive confirmed with DFA or another LCR assay with a different DNA target. Thus it appears that molecular amplification techniques for the detection of *C. trachomatis* in urine specimens from both men and women are a substantial improvement over conventional diagnostic and screening methods and will provide an important tool for decreasing the reservoir of infection, especially in asymptomatic men.

In the diagnostic laboratory, molecular techniques present different problems for specimen handling and interpretation of results than cell culture or antigen detection (15). Inherent in the increased sensitivity of these molecular techniques is the potential for false-positive results due to cross contamination between specimens, and run-to-run contamination from equipment, reagents, and supplies. These problems can be overcome by observing stringent rules for specimen preparation (e.g., dedicated equipment) and separating specimen processing and reagent preparation areas to prevent contamination. Enzymatic or photochemical sterilization can be used to eliminate run-to-run contamination. False-negative results may be due to substances in specimens inhibitory to enzymes used for amplification. Known inhibitors include phosphate ions, heparin, heme, crystals in the urine specimens, and detergents used in specimen processing. Internal controls are now commercially available to detect false negatives.

Although molecular tests are more expensive than EIA, cost-effectiveness studies should take into consideration the benefits of averting the enormous costs of long-term reproductive sequelae in women with undetected infections, adverse pregnancy outcomes, and HIV infection. Targeted screening of women to detect cervical chlamydial infection decreases the incidence of symptomatic PID (23). Patients with genital gonococcal or chlamydial infections are also at increased risk for human immunodeficiency virus (HIV) (24). Although the risk for HIV may be lower in patients with chlamydial infection than in those with genital ulcer disease, the higher prevalence of chlamydial infection in some populations means that the population attributable risk for HIV may be substantially higher for chlamydia. Shortening the duration of infectiousness by early diagnosis and treatment could have a major impact on risk reduction for HIV infection. A recent study showed that strengthening sexually transmitted disease control through education, access to diagnosis, and treatment reduced the incidence of HIV by 42% in study communities in Tanzania over 2 years (25).

### Treatment

Azithromycin prescribed as a single oral 1-g dose is equivalent to the traditional 7-day regimen of doxycycline for treating ocular and uncomplicated genital chlamydial infections (26-28). Compared with conventional therapy, azithromycin has excellent pharmacokinetic characteristics, such as increased bioavailability; lower incidence of gastrointestinal tract side effects; and increased concentration in mucus, macrophages, and tissues with a half life of 5 to 7 days (29). These characteristics allow for single dosing, which alleviates the problem of patient noncompliance with multiday regimens. With single-dose therapy, the potential for reinfection due to earlier resumption of sexual activity is a concern. At present, there are limited data on the use of single-dose therapy in adolescents, during pregnancy, and for syndromes such as PID, cervicitis, and nongonococcal urethritis (30-33). Studies are needed to determine if these regimens achieve clinical and microbiologic cure while preserving fertility and preventing further tissue damage to the upper genital tract.

Although the higher cost of azithromycin may be prohibitive for its use in resource-limited settings, selective use in persons at high risk or in those with a history of noncompliance may prove cost-effective. The cost of retreatment as a result of noncompliance and the additional cost of contact tracing can make single dose azithromycin more cost-effective than doxycycline (34).

### Pathogenesis

Interesting findings in three areas of *C. trachomatis* pathogenesis further delineate the complex bacteria-host relationship in disease and may have implications for vaccine design. These new observations include the extensive but unexpected polymorphism of the major outer membrane protein (MOMP), the evidence for genetic susceptibility to disease, and the association of antibody response to the 60 kDa heat shock protein (CHSP60) with the development of adverse sequelae following ocular and genital infections.

### Polymorphism of MOMP

The ecologic success of a pathogen is determined in part by its ability to evade host defenses. With *C. trachomatis*, MOMP is a major target for protective host immune responses, such as neutralizing antibodies and possibly, protective T-cell responses (35,36). The basis for MOMP antigenic variation is allelic polymorphism at the omp-1 locus, and immune selection appears to be occurring in host populations frequently exposed to *C. trachomatis* (37). Each variant apparently only infects hosts lacking serovar-specific immunity to that variant, and the ecologic success of chlamydiae may be due to their ability, under immune selection pressure, to generate successive allelic variants (36). DNA sequence analyses of isolates from different populations show that most MOMP variants are results of single amino acid substitutions (37-39). Recombination of sequences from MOMP during mixed infections may also have occurred. Recombinant variants with mosaic sequences of MOMP from different strains were especially frequent in persons with high rates of infection. MOMP variants were also more frequently found in women with PID than in those with lower genital tract infections, which suggests a relationship between sequence variation in MOMP and more invasive disease (39). Clearly, the extensive polymorphism of MOMP, the tempo for variation, and the mechanism of immune selection have important implications for vaccine design (35).

### Genetic Susceptibility to Disease

HLA B27 has been associated with Reiter's syndrome following genital chlamydial infection (40). Only a subset of infected persons appear to have long-term complications after acute or repeated chlamydial infections. In a study of 306 persons from trachoma-endemic communities in the Gambia, the HLA class I antigen HLA-A28 was significantly more common in case-patients than in age-, sex-, and location-matched controls (41). In particular, the A*6802 allele was overrepresented among case-patients. It may be that immunopathology is associated with HLA-A*6802 restricted cytotoxic T-lymphocyte responses. The frequency of HLA class II alleles was similar among cases and controls suggesting that, if class II restricted T-cell responses are important in immunopathology, they were not targeted at single epitopes. No individual HLA type was associated with protection from scarring, which suggests that multiple or complex T-cell responses may be involved in protective immunity. Susceptibility to chlamydial PID in a study of sex workers in Nairobi, Kenya, has been associated with a HLA class I allele, HLA A-31 (42). Studies are needed to determine whether susceptibility to silent PID, ectopic pregnancy, and progression to tubal factor infertility are associated with HLA class I restricted immune responses.

### Role of CHSP60 in Immunopathology

Antibody response to a 57 kDa chlamydial protein was initially observed more frequently in women with tubal infertility than in controls (43). This protein was subsequently identified as a heat shock protein of the GroEL family of stress proteins. The association between antibody response to CHSP60 and PID, ectopic pregnancy, tubal infertility, and trachoma (44-48) has been documented. The risk factors associated with CHSP60 antibody response are similar to those for chlamydial PID and include older age and chronic or repeated infections. There appears to be genetic restriction for the CHSP60 antibody response. In a study of trachoma in the Gambia, HLA DRB1*0701 was positively correlated with CHSP60 response, while DRB1*0301 and DQB1*0501 were negatively associated (48). However, these alleles were not associated with trachoma and may reflect linkage disequilibrium between HLA class II alleles and polymorphic markers for other immune response genes.

At present, it remains unclear whether antibody to CHSP60 is causally involved in chlamydial immunopathogenesis or is merely a marker of persistent chlamydial infection (35). Both may be true. In cells persistently infected with *C. trachomatis*, the expression of CHSP60 is normal, while other antigens, such as MOMP, are downregulated, thus providing continued antigenic stimulation for the CHSP60 antibody response observed in persons with long-term sequelae (49). T-cell responses to chlamydial antigens, including CHSP60, were more depressed in persons with trachoma than in those who recovered from infection without sequelae (50). Persons with trachoma or reproductive sequelae have high levels of serum antibody response to *C. trachomatis*. In guinea pigs and in gene knock-out mice, both B- and T-cell responses have been important in immunity and resolution of infection (51,52). Therefore, persons with long-term sequelae may have predominantly $Th_2$ responses, characterized by high levels of B-cell response and inadequate T-cell responses that may not clear the infection thus leading to chronic inflammation. Immunopathology may also be the result of a hit-and-run mechanism in which immune response to CHSP60 breaks self-tolerance to the human HSP60 and leads to an autoimmune reaction that results in tissue damage (35).

### *C. psittaci*

### Epidemiology

Human infections with *C. psittaci* are caused by occupational exposure to infected birds or household handling of nasal discharge or fecal material from pet birds. Birds can be healthy carriers of *C. psittaci*. Increased shedding and susceptibility to disease occur under conditions of stress such as shipping, crowding, starvation, or egg laying. Person-to-person transmission is rare but has been observed in outbreaks. In the *C. psittaci* pandemic of 1929-30, infected birds from Argentina were shipped to different parts of the world causing outbreaks of infection worldwide with death rates of up to 40% (5). Since then *C. psittaci* has been isolated from more than 130 species of birds. Thus, all avian species, including wild birds, should be regarded as potential sources of zoonosis.

Reports of outbreaks of psittacosis in duck and turkey processing plants show that, in spite of availability of medicated feed, diagnostic testing, and screening of poultry, *C. psittaci* infections continue to be a public health concern (53,54). High rates of chlamydial infection in household cats and asymptomatic carriage of *C. psittaci* in cats from breeding catteries raise the possibility that human *C. psittaci* infection from pets other than birds may be underdiagnosed (6,7,55,56). Studies of animal and cellular tropism of various strains within the species may give important clues to the pathogenesis of *C. psittaci* infections.

### Clinical Manifestations

Human infection caused by exposure to infected birds or poultry is manifested as a flulike illness characterized by fever, chills, headache, and less frequently, cough, myalgias, rash, arthralgia and joint swelling, and atypical pneumonia in more severe cases. The incubation period is 6 to 19 days. Infections transmitted from ruminants are rare, but placentitis, disseminated intravascular coagulation, and spontaneous abortion in women exposed to infected sheep during lambing have been reported (56). Zoonoses associated with exposure to ruminants are characterized by multiorgan involvement often resulting in hepatic and renal dysfunction and endocarditis. Human conjunctivitis, glomerulonephritis, and endocarditis caused by *C. psittaci* from infected cats and pigeons have been reported (55).

### Diagnosis and Treatment

Serodiagnosis has been the method of choice for human *C. psittaci* infections because culture is technically demanding and represents an important biohazard. The complement fixation assay is genus specific. Its interpretations should depend on clinical symptoms and patient history. The microimmunofluorescence (MIF) assay can detect species-specific IgM or IgG antibodies. Antigen detection methods, such as EIA, have been used, but they are based on the capture of the genus-specific LPS. PCR assays are not yet commercially available but can offer lower detection limits of 10 EBs or less (57,58). Molecular techniques not only provide more sensitive and rapid diagnosis than serology, but they also provide the opportunity for fingerprinting strains. This is particularly useful in outbreak investigations and for the confirmation of zoonotic transmission from infected birds or animals.

The recommended treatment for *C. psittaci* infection is 250 mg of tetracycline 4 times daily for 21 days. Although the death rate is low, prolonged hospitalization may be required. Protracted recovery and high incidence of relapse have also been noted.

### *C. pneumoniae*

### Epidemiology

*C. pneumoniae* is a common cause of acute respiratory tract infections and accounts for 6% to 10% of community-acquired pneumonia (8). Infection is usually mild or asymptomatic but can be severe, especially in the elderly, probably as a result of underlying illness, impaired mucociliary clearance, and immune senescence. Unlike *C. psittaci*, *C. pneumoniae* is spread by person-to-person transmission by respiratory droplet and has an incubation period of 7 to 21 days. Outbreaks of infection have been reported in families, schools, military barracks, and nursing homes. Coinfection with viruses (e.g., influenza and respiratory syncytial virus) and with bacteria has been reported frequently. Seroepidemiologic studies show that most primary infections occur during school age and the early teenage years; among adults seroprevalence is 40% to 70%. Reinfections are common, and serum antibodies do not appear to be protective.

### Laboratory Diagnosis

Accurate and rapid laboratory diagnostic methods leading to improved patient care, appropriate use of antimicrobial therapy, and better understanding of the epidemiology of this emerging pathogen (59,60) are needed. Culture is highly specific but is technically demanding often requiring multiple passages over a period of weeks to show a positive result. *C. pneumoniae* has been isolated from the nasopharynx of healthy persons, but the rate of asymptomatic carriage in a normal population is unknown (61).

Antigen detection tests, such as EIA and DFA, and molecular detection methods, such as PCR assays, provide a rapid diagnosis without stringent transport requirements. Monoclonal antibodies specific for *C. pneumoniae* are now commercially available for DFA and for culture confirmation (62). PCR assays have lower detection limits of 10 to 100 EBs (57,58,63-65). The protocol developed by Tong and Sillis amplifies a target sequence conserved between *C. pneumoniae* and *C. psittaci* and hence can detect DNA from either pathogen in a single assay (57). A nested PCR procedure is used to differentiate between the *C. pneumoniae* and *C. psittaci* amplicons. The protocol of Rasmussen et al. amplifies a genus-specific target, followed by species differentiation using restriction enzyme digestion (58). The development of multiplex PCR assays containing primers specific for a panel of respiratory pathogens will be useful.

The MIF assay is the standard method used for chlamydia serology today. Ekman compared the performance of the complement fixation (CF), LPS-based EIA, and MIF tests for the serodiagnosis of *C. pneumoniae* and *C. psittaci* infections in an elderly population and found that the CF test has a sensitivity of 10% compared with 88% and 72% for MIF and EIA, respectively (66). IgM antibodies were only detected in 11% of the cases. IgM antibodies are rarely produced in reinfections with *C. pneumoniae.* CF tests may be useful in early initial infections as LPS antibodies are produced early in infection. Serodiagnosis may be made by demonstrating a fourfold rise in CF or EIA titer in paired sera taken a week apart, compared with the 3 weeks or more that it takes by MIF to demonstrate seroconversion. Because reinfections are common and LPS-based serologic tests are not useful in reinfection, the MIF assay remains the most useful and specific tool for the serodiagnosis of respiratory infections due to *C. pneumoniae.*

### Treatment

The newer macrolides, clarithromycin and azithromycin, with longer tissue half-life and concentration in mucus and macrophages and improved bioavailability can potentially provide shorter and better-tolerated regimens for the treatment of respiratory infections due to *C. pneumoniae* than doxycycline or erythromycin, which have to be given for 2 to 3 weeks to avoid relapse. They may also be preferred for empiric therapy as they provide broader coverage than erythromycin against etiologic agents in community-acquired pneumonia. The optimal duration of treatment for respiratory infections due to *C. pneumoniae* needs to be determined since studies with documented microbiologic cure are limited, and recurrence of infection is common (67).

### Association with Atherosclerosis

The association of *C. pneumoniae* infection with coronary heart disease and acute myocardial infarction was first made on the basis of elevated IgG and IgA antibodies and LPS containing immune complexes in 50% to 60% in patients with coronary heart disease or acute myocardial infarction compared with 7% to 12% in the controls. This study did not take into account risk factors for heart disease such as smoking, hypertension, or serum lipid levels. Subsequently, several cross-sectional studies involving 46 to 461 study participants have shown that a similar association of IgG antibodies against *C. pneumoniae* with coronary artery disease and carotid disease with adjusted odds ratios of 1.6 to 2.6 after controlling for known risk factors (68-72). Electron microscopy, PCR, and immunochemical evidence of *C. pneumoniae* in coronary arterial fatty streaks and atheromatous plaques have also been described (72,73).

Two more recent studies reported equivocal findings. In one, *C. pneumoniae* was detected in 79% of 90 coronary atherectomy specimens from symptomatic patients by direct immunofluorescence and was confirmed by electron microscopy. Only 4% of 24 control nonatherosclerotic coronary specimens were positive for *C. pneumoniae* (74). The 24 control samples included 12 from heart transplant patients whose arteries were damaged, but not by atherosclerosis. The absence of *C. pneumoniae* in these tissue samples argues against its role as a passenger recruited to the site of injury in macrophages. In the other study, *C. pneumoniae* was not detected in 58 coronary atheroma specimens by culture, PCR, or electron microscopy (75). The seroprevalence of *C. pneumoniae* in 65 case-patients was not different from that in 28 asymptomatic controls. In fact, IgG titers were higher in controls than in case-patients. Nonetheless, data suggest that the association of *C. pneumoniae* with atherosclerosis is consistent and biologically plausible. Whether *C. pneumoniae* is causally involved or is a bystander trapped in the atherogenic process is unclear.

The sustained IgA and IgG antibody levels against *C. pneumoniae* in persons with atherosclerosis suggest that chronic infection may be frequent after infection. The site of colonization for a chronic *C. pneumoniae* infection may be in the alveolar macrophages of the lung. Thus the initial event in atherogenesis may be the formation of the fatty streak. Fatty streaks consist of lipid-laden macrophages derived from blood monocytes and T lymphocytes attracted to the arterial subintima. Conversion of the fatty streak to atheroma depends on many factors, e.g., the proliferation and differentiation of smooth muscle cells and fibroblasts. Chronic infection with *C. pneumoniae* may result from organisms harbored in macrophages trapped in the arterial wall. Growth of *C. pneumoniae* in endothelial, smooth muscle cells, and macrophages from peripheral blood monocytes has been reported (76). Injured blood vessels initiate events that promote thrombosis and platelet adhesion at the site of injury. These events in turn promote atherosclerosis. Tissue injury through *C. pneumoniae*-specific circulating immune complexes in patients with chronic heart disease may be an alternate mechanism or compounding atherogenesis. The idea that an infectious agent is involved in the atherogenic process is not new, but the role of *C. pneumoniae* in this process needs to be defined.

### Association with Asthma

The prevalence of asthma, an important chronic respiratory disorder, has been steadily increasing. Viral and *Mycoplasma pneumoniae* infections have been implicated in exacerbating the disease. The first observations on the association of *C. pneumoniae* infection with the exacerbation of asthma were made in 1986 when wheezing was associated with acute bronchitis due to *C. pneumoniae* infection (8,77). Subsequent studies showed that exacerbation of asthma due to *C. pneumoniae* infection may occur in 1% to 11% of respiratory infections in adults as well as children. The mechanism underlying the association is unclear. Preliminary results in animal models suggest that *C. pneumoniae* can produce persistent infection and cause pulmonary inflammation, and production of chlamydia-specific IgE antibodies in children with reactive airway disease has been demonstrated (78). A possible scenario for this association is an antigen-specific allergic reaction with the release of pulmonary inflammatory mediators and recruitment of inflammatory cells to the airways, causing airway epithelial damage. Activated T lymphocytes and cytokines appear to play a critical role as mediators of persistent inflammation in asthma. IL-4 is essential for B lymphocytes class switching from IgG to IgE. In vitro human IgE synthesis is reciprocally regulated by IL-4 and interferon-gamma. Thus cytokines from a $Th_2$ response to infection would facilitate and promote IgE production. Immunotherapy or glucocorticoid therapy targeting $CD4^+$ T cells may decrease the proinflammatory role of these cells and alleviate symptoms of asthma. The role of persistent infection in the pathogenesis of asthma merits further study because, unlike viral infections, *C. pneumoniae* infections can be eradicated through appropriate antimicrobial therapy.

The hallmark of chlamydial infection is that most persons infected have mild to no apparent clinical disease and some have severe disease. Asymptomatic infection not only creates a problem in detecting cases for disease control programs but also contributes to the development of long-term adverse sequelae, such as scarring trachoma from ocular *C. trachomatis* infection, pelvic inflammatory disease, ectopic pregnancy, and tubal factor infertility from genital *C. trachomatis* infection. The recent availability of effective single dose oral antimicrobial therapy and sensitive molecular amplification tests that allow the use of noninvasive specimens for diagnosis and screening is expected to have a major impact in reducing the prevalence of disease in the next decade. New information from cell biology as well as data from observing the interaction of chlamydiae with the host in terms of metabolic requirements and immune evasion strategies offer clues about the pathogenesis of chlamydia infections and may eventually lead to an effective vaccine. Sporadic outbreaks of psittacosis continue to be reported despite the use of medicated feed and the screening of poultry. Recent reports of *C. psittaci* in cats from breeding catteries illustrate the potential of zoonotic diseases transmissible to humans from pets other than birds. Two new species of chlamydiae, *C. pneumoniae* and *C. pecorum*, were designated in 1989 and 1992, respectively. Clinical manifestations associated with *C. pneumoniae* infection continue to emerge. Possible links to chronic conditions, such as atherosclerosis and asthma remain to be elucidated. With the recent discovery of the involvement of infectious agents in other chronic conditions, it seems reasonable to apply molecular tools for chlamydial detection to identify their potential involvement in other etiologically undefined chronic inflammatory conditions such as inflammatory bowel disease and rheumatoid arthritis.

*Dr. Peeling is a research scientist and chief of the Division of Chlamydial and Mycoplasma Diseases at the Laboratory Centre for Disease Control, Health Canada. She is interested in the diagnosis and pathogenesis of chlamydial infections with particular emphasis on the development, risk assessment, and possible prevention of adverse ocular and reproductive sequelae in human chlamydial infections.*

*Dr. Brunham is professor and head of the Department of Medical Microbiology at the University of Manitoba. He has a long standing interest in the immunology of chlamydial infections, and his current research focus is on vaccine development.*

### References

1. Weisburg WG, Hatch TT, Woese CR. Eubacterial origin of chlamydiae. J Bacteriol 1986;167:570-4.
2. Dawson CR, Jones BR, Tarizzo ML. Guide to trachoma control in programmes for the prevention of blindness. Geneva: World Health Organization, 1981.
3. Sexually Transmitted Diseases. World Health Organization Press Release WHO/64, 25 August 1995.
4. Washington A, Johnson RE, Sanders L Jr. *Chlamydia trachomatis* infection in the United States: what are they costing us? JAMA 1987;257:2070-2.
5. Schachter J, Dawson CR. Psittacosis. Human Chlamydial Infections. Littleton, MA: PSG Publishing Co., 1978.
6. Nasisse MP, Guy JS, Stevens JB, English RV, Davidson MG. Clinical and laboratory findings in chronic conjunctivitis in cats: 91 cases (1983-1991). J Am Vet Med Assoc 1993;203:834-7.
7. Pointon AM, Nicholls JM, Neville S, Allanson M, Coles C, Lawrence D. Chlamydia infection among breeding catteries in south Australia. Australian Veterinary Practitioner 1991;21:58-63.
8. Grayston JT. Infections caused by *Chlamydia pneumoniae* strain TWAR. Clin Infect Dis 1992;15:757-63.
9. Stephens RS. Molecular mimicry and *Chlamydia trachomatis* infection of eucaryotic cells. Trends Microbiol 1994;2:99-101.
10. Hackstadt T, Scidmore MA, Rocky DD. Lipid metabolism in *Chlamydia trachomatis*-infected cells: directed trafficking of Golgi-derived sphingolipids to the chlamydial inclusion. Proc Natl Acad Sci U S A 1995;92:4877-81.
11. Hackstadt T, Rocky DD, Heinzen RA, Sidmore MA. *Chlamydia trachomatis* interrupts an exocytic pathway to acquire endogenously synthesized sphingomyelin in transit from the Golgi apparatus to the plasma membrane. EMBO J 1996;15:964-77.
12. McClarty G. Chlamydiae and the biochemistry of intracellular parasitism. Trends Microbiol 1994;2:157-64.

13. Peeling RW. *Chlamydia trachomatis* and *Neisseria gonorrhoeae*: Pathogens in retreat? Current Opinion in Infectious Diseases 1995;8:26-34.
14. Lin JSL, Jones WE, Yan L, Wirthwein KA, Flaherty EE, Haivanis RM, et al. Underdiagnosis of *Chlamydia trachomatis* infection: diagnostic limitations in patients with low-level infection. Sex Transm Dis 1992;19:259-65.
15. Peeling RW, Brunham RC. Molecular techniques for the laboratory identification of *Chlamydia trachomatis.* Journal of the International Federation of Clinical Chemistry 1994;6:78-82.
16. Bauwens JE, Clark AM, Stamm WE. Diagnosis of *Chlamydia trachomatis* endocervical infections by a commercial polymerase chain reaction assay. J Clin Microbiol 1993;31:3023-7.
17. de Barbeyrac B, Pellet I. Dutilh B, Bebear C, Dumon B, Geniaux M, et al. Evaluation of the Ampicor *Chlamydia trachomatis* test versus culture in genital samples in various prevalence populations. Genitorurin Med 1994;70:162-6.
18. Wisenfeld HC, Uhrin M, Dixon BW, Sweet RL. Diagnosis of male *Chlamydia trachomatis* urethritis by polymerase chain reaction. Sex Transm Dis 1994;21:268-71.
19. Jaschek G, Gaydos CA, Welsh L, Quinn TC. Direct detection of *Chlamydia trachomatis* in urine specimens from symptomatic and asymptomatic men by using a rapid polymerase chain reaction assay. J Clin Microbiol 1993;31:1209-12.
20. Toye B, Peeling RW, Jessamine P, Claman P, Gemmill I. Diagnosis of *Chlamydia trachomatis* infections in asymptomatic men and women by PCR assay. J Clin Microbiol 1996;34:1396-400.
21. Chernesky MA, Jang D, Lee H, Hu H, Sellors J, Tomazic-Allen SJ, et al. Diagnosis of *Chlamydia trachomatis* infections in men and women by testing first-void urine by ligase chain reaction. J Clin Microbiol 1994;32:2682-5.
22. Schachter J, Stamm WE, Quinn TC, Andrews WW, Burczak JD, Lee H. Ligase chain reaction to detect *Chlamydia trachomatis* infection of the cervix. J Clin Microbiol 1994;32:2540-3.
23. Scholes D, Stergachis A, Heidrich FE, Andrilla HA, Holmes KK, Stamm WE. Selective screening for chlamydia reduces the incidence of pelvic inflammatory disease: results from a randomised intervention trial. J Infect Dis 1996 (in press).
24. Cameron DW, Simonsen JN, D'Costa LJ, Ronald AR, Maitha GM, Gakinya MN, et al. Female to male transmission of human immunodeficiency virus type 1: risk factors for seroconversion in men. Lancet 1989;ii:403-7.
25. Grosskurth H, Mosha F, Todd J, Mwijarubi E, Klokke A, Senkoro K, et al. Impact of improved treatment of sexually transmitted diseases on HIV infection in rural Tanzania: randomised controlled trial. Lancet 1995:346:530-6.
26. Martin DH, Mroczkowski TF, Dalu ZA, McCarty J, Jones RB, Hopkins SJ, et al. A controlled trial of a single dose of azithromycin for the treatment of chlamydial urethritis and cervicitis. N Engl J Med 1992;327:921-5.
27. Bailey RL, Arullendran P, Whittle HC, Mabey DCW. Randomised controlled trial of single-dose azithromycin in the treatment of trachoma. Lancet 1993;342:453-6.
28. Ossewaarde JM, Plantema FHF, Rieffe M, Nawrocki RP, de Vries A, van Loon AM. Efficacy of single-dose azithromycin versus doxycycline in the treatment of cervical infections caused by *Chlamydia trachomatis.* Eur J Clin Microbiol Infect Dis 1992;11:693-7.
29. Worm A-M, Osterlind A. Azithromycin levels in cervical mucus and plasma after a single 1.0 g oral dose for chlamydia cervicitis. Genitourin Med 1995;71:244-6.
30. Hammerschlag MR, Golden NH, Oh MK, Gelling M, Sturdevant M, Pernell PR, et al. Single dose of azithromycin for the treatment of genital chlamydial infections in adolescents. J Pediatr 1993;122:961-5.
31. Bush MR, Rosa C. Azithromycin and erythromycin in the treatment of cervical chlamydial infection during pregnancy. Obstet Gynecol 1994;84:61-3.
32. Lauharanta J, Saarinen K, Mustonen M-T, Happonen H-P. Single-dose oral azithromycin versus seven-day doxycycline in the treatment of non-gonococcal urethritis in males. J Antimicrob Chemother 1993;31:177-83.
33. Lister PJ, Balechandran T, Ridgway GL, Robinson JA. Comparison of azithromycin and doxycycline in the treatment of non-gonococcal urethritis in men. J Antimicrob Chemother 1993;31:185-92.
34. Magid D, Douglas JM, Schwartz JS. Doxycycline compared with azithromycin for treating women with genital *Chlamydia trachomatis* infections: an incremental cost-effectiveness analysis. Ann Intern Med 1996;124:389-99.
35. Brunham RC, Peeling RW. Chlamydia trachomatis antigens: role in immunity and pathogenesis. Infect Agents Dis 1994;3:218-33.
36. Brunham RC, Plummer F, Stephens RS. Bacterial antigenic variation, host immune response and pathogen-host co-evolution. Infect Immun 1993;61:2273-6.
37. Brunham R, Yang C, Maclean I, Kimani J, Maitha G, Plummer F. *Chlamydia trachomatis* from individuals in a sexually transmitted disease core group exhibit frequence sequence variation in the major outer membrane protein (omp1) gene. J Clin Invest 1994;94:458-63.
38. Hayes LJ, Bailey RL, Mabey DCW, Clarke IN, Pickett MA, Watt PJ, et al. Genotyping of *Chlamydia trachomatis* from a trachoma endemic village in the Gambia by a nested polymerase chain reaction: identification of strain variants. J Infect Dis

1992;166:1173-7.
39. Dean D, Schachter J, Dawson CR, Stephens RS. Comparison of the major outer membrane protein variant sequence regions of B/Ba isolates: a molecular epidemiologic approach to *Chlamydia trachomatis* infections. J Infect Dis 1992;166:383-92.
40. Schachter J, Dawson CR. Reiter's Syndrome. Human Chlamydial Infections. Littleton, MA: PSG Publishing Co., 1978.
41. Conway DJ, Holland MJ, Campbell AE, Bailey RL, Krausa P, Peeling RW, et al. HLA class I and class II polymorphism and trachomatous scarring in a chlamydia trachomatis-endemic population. J Infect Dis 1996;174:643-6.
42. Kimani J, Maclean IW, Bwayo JJ, MacDonald K, Oyugi J, Maitha GM, et al. Risk factors for Chlamydia trachomatis pelvic inflammatory disease among sex workers in Nairobi, Kenya. J Infect Dis 1996;173:1437-44.
43. Brunham RC, Maclean IW, Binns B, Peeling RW. *Chlamydia trachomatis*: its role in tubal infertility. J Infect Dis 1985;152:1275-82.
44. Wagar EA, Schachter J, Bavoil P, Stephens RS. Differential human serologic response to two 60,000 molecular weight *Chlamydia trachomatis* antigens. J Infect Dis 1990;162:922-7.
45. Brunham RC, Peeling R, Maclean I, Kosseim ML, Paraskevas M. *Chlamydia trachomatis*-associated ectopic prgnancy:serologic and histologic correlates. J Infect Dis 1992;165:1076-81.
46. Toye B, Laferriere C, Claman P, Jessamine P, Peeling R. Association between antibody to the chlamydial heat shock protein and tubal infertility. J Infect Dis 1993;168:1236-40.
47. Arno JN, Yuan Y, Cleary RE, Morrison RP. Serologic responses of infertile women to the 60-kd chlamydial heat shock protein. Fertil Steril 1995;64:730-5.
48. Peeling RW, Bailey RL, Conway D, Holland MJ, Dillon E, Mabey DCW. Antibody response to the chlamydial heat shock protein 60 is associated with scarring trachoma. 96th American Society for Microbiology meeting, New Orleans, May 1996. Abstract #2871.
49. Beatty WL, Byrne GI, Morrison RP. Morphologic and antigenic characterization of interferon gamma mediated persistent *Chlamydia trachomatis* infection in vitro. Proc Natl Acad Sci U S A 1993;90:3998-4002.
50. Holland MJ, Bailey RL, Hayes LJ, Whittle HC, Mabey DCW. Conjunctival scarring in trachoma is associated with depressed cell-mediated immune responses to chlamydial antigens. J Infect Dis 1993;168:1528-31.
51. Morrison RP, Feilzer K, Tumas DB. Gene knock-out mice establish a primary role for major histocompatibility complex class II-restricted responses in Chlamydia trachomatis genital tract infection. Infect Immun 1995;63:4661-8.
52. Igietseme JU, Magee DM, Williams DM, Rank RG. Role for $CD8^+$ T cells in antichlamydial immunity defined by chlamydia-specific T-lymhocyte clones. Infect Immun 1994;62:5195-7.
53. Hinton DG, Shipley A, Galvim JW, Harkin JT, Brunton RA. Chlamydiosis in workers at a duck farm and processing plant. Aust Vet J 1993;70:174-6.
54. Hedberg K, White KE, Forfang JC, Korlath JA, Friendshuh, Hedberg CW, et al. An outbreak of psittacosis in Minnesota turkey industry workers: implications for modes of transmission and control. Am J Epidemiol 1989;130:569-77.
55. Hadley KM, Carrington D, Frew CE, Gibson AAM, Hislop WS. Ovine chlamydiosis in an abattoir worker. J Infect Dis 1992;25:105-9.
56. Regan RJ, Dathan JRE, Treharne JD. Infective endocarditis with glomerulonephritis associated with cat chlamydia (*C. psittaci)* infection. British Heart Journal 1979;42:349-52.
57. Tong CYW, Sillis M. Detection of *Chlamydia pneumoniae* and *Chlamydia psittaci* in sputum samples by PCR. J Clin Pathol 1993;46:313-7.
58. Rasmussen SJ, Douglas FP, Timms P. PCR detection and differentiation of *Chlamydia pneumoniae, Chlamydia psittaci* and *Chlamydia trachomatis.* Mol Cell Probes 1992;6:389-94.
59. Saikku P. Diagnosis of acute and chronic *Chlamydia pneumoniae* infections. In: Orfila J, et al., editors. Chlamydial Infections. Bologna: Societa Editrice Esculapio, 1994.
60. Peeling RW. Laboratory diagnosis of *Chlamydia pneumoniae* infections. Canadian Journal of Infectious Diseases 1995;6:198-203.
61. Gnarpe J, Gnarpe H, Sundelof B. Endemic prevalence of *Chlamydia pneumoniae* in subjectively healthy persons. Scand J Infect Dis 1991;23:387-8.
62. Montalban GS, Roblin PM, Hammerschlag MR. Performance of three commercially available monoclonal reagents for confirmation of *Chlamydia pneumoniae* in cell culture. J Clin Microbiol 1994;32:1406-7.
63. Campbell LA, Melgosa MP, Hamilton DJ, Kuo C-C, Grayston JT. Detection of *Chlamydia pneumoniae* by polymerase chain reaction. J Clin Microbiol 1992;30:434-9.
64. Gaydos CA, Roblin PM, Hammerschlag MR, Hyman CL, Eiden JJ, Schacter J, et al. Diagnostic utility of PCR-Enzyme Immunoassay, culture, and serology for the detection of *Chlamydia pneumoniae* in symptomatic and asymptomatic patients. J Clin Microbiol 1994;32:903-5.
65. Black CM, Fields PI, Messmer TO, Berdal BP. Detection of *Chlamydia pneumoniae* in clinical specimens by polymerase chain reaction using nested primers. Eur J Clin Microbiol Infect Dis 1994;13:752-6.
66. Ekman MR, Leinonen M, Syrjala H, Linnanmaki E, Kujala P, Saikku P. Evaluation of serological methods in the diagnosis of *Chlamydia pneumoniae* during an epidemic in Finland. Eur J Clin Microbiol Infect Dis 1993;12:756-60.

67. Roblin P, Montalban G, Hammerschlag MR. Susceptibilities to clarithromycin and erythromycin of isolates of *Chlamydia pneumoniae* from children with pneumonia. Antimicrob Agents Chemother 1994;38:1588-9.
68. Grayston JT, Thom DH, Kuo C-C, Campbell LA, Wang S-P. *Chlamydia pneumoniae* (TWAR) and atherosclerosis. In: Orfila J, Byrne G, Chernesky MA, et al, editors. Chlamydial Infections. Bologna: Societa Editrice Esculapio, 1994.
69. Saikku P, Leinonen M, Tenkanen L, Linnanmaki E, Ekman M-R, Manninen V, et al. Chronic *Chlamydia pneumoniae* Infection as a Risk Factor for Coronary Heart Disease in the Helsinki Heart Study. Ann Intern Med 1992;116:273-8.
70. Thom D, Grayston JT, Siscovick D, Wang S-P, Weiss N, Daling J. Association of Prior Infection With *Chlamydia pneumoniae* and Angiographically Demonstrated Coronary Artery Disease. JAMA 1992;268:68-72.
71. Melnick S, Shahar E, Folsom A, Grayston JT, Sorlie P, Wang S-P, et al. Past Infection by *Chlamydia pneumoniae* Strain TWAR and Asymptomatic Carotid Atherosclerosis. Am J Med 1993;95:499-504.
72. Shor A, Kuo CC, Patton D. Detection of *Chlamydia pneumoniae* in coronary arterial fatty streaks and atheromatous plaques. S Afr Med J 1992;82:158-61.
73. Kuo CC, Shor A, Campbell L, Fukushi H, Patton D, Grayston JT. Demonstration of *Chlamydia pneumoniae* in Atherosclerotic Lesions of Coronary Arteries. J Infect Dis 1993;167:841-9.
74. Muhlestein JB, Hammond E, Carlquist JF, Radicke E, Thomson MJ, Karagounis LA, et al. Increased incidence of *Chlamydia* species within the coronoary arteries of patients with symptomatic atherosclerotic versus other forms of cardiovascular disease. J Am Coll Cardiol 1996;27:1555-61.
75. Weiss SM, Roblin PM, Gaydos C, Cummings P, Patton D, Schulhoff N, et al. Failure to detect *Chlamydia pneumoniae* in coronary atheromas of patients undergoing atherectomy. J Infect Dis 1996;173:957-62.
76. Godzik K, O'Brien E, Wang S-K, Kuo CC. In Vitro Susceptibility of Human Vascular Wall Cells to Infection with *Chlamydia pneumoniae*. J Clin Microbiol 1995;33:2411-4.
77. Hahn DL, Dodge RW, Golubjatnikov R. Association of *Chlamydia pneumoniae* (TWAR) infection with wheezing, asthmatic bronchitis and adult-onset asthma. JAMA 1991,266:225-30.
78. Emre U, Sokolovskaya N, Roblin PM, Schachter J, Hammerschlag M. Detection of anti-*Chlamydia pneumoniae* IgE in children with reactive airway disease. J Infect Dis 1995;148:727-32.
